# THAP and ATF-2 Regulated Sterol Carrier Protein-2 Promoter Activities in the Larval Midgut of the Yellow Fever Mosquito, *Aedes aegypti*

**DOI:** 10.1371/journal.pone.0046948

**Published:** 2012-10-04

**Authors:** Rong Peng, Qiang Fu, Huazhu Hong, Tyler Schwaegler, Que Lan

**Affiliations:** 1 College of Life Sciences, Central China Normal University, Wuhan, Hubei, China; 2 Department of Entomology, University of Wisconsin, Madison, Wisconsin, United States of America; New Mexico State University, United States of America

## Abstract

Expression of sterol carrier protein-2 (*SCP-2*) in *Aedes aegypti* shows a distinct temporal/spatial pattern throughout the life cycle. In order to identify the transcription factors responsible for the larval temporal/spatial regulation of *AeSCP-2* transcription, *AeSCP-2* promoter activities were studied *in vivo* via transient transfection of promoter/reporter gene assays. Regulatory sequences upstream −1.3 kb of the transcription start site of *AeSCP-2* were found to be critical for the *in vivo* temporal/spatial promoter activity. Interestingly, the −1.6 kb promoter sequence efficiently drove the larval midgut-specific siRNA expression, indicating that the −1.6 kb upstream sequence is sufficient for temporal/spatial *AeSCP-2* transcriptional activity. Four transcription factors were identified in the midgut nuclear extract from feeding larvae via labeled −1.6/−1.3 kb DNA probe pull-down and proteomic analysis. Co-transfection of the promoter/reporter gene with inducible siRNA expression of each transcription factor was performed to confirm the regulatory function of individual transcription factor on *AeSCP-2* transcriptional activities in the larval midgut. The results indicate that two of the identified transcription factors, Thanatos-associated protein (THAP) and activating transcription factor-2 (ATF-2), antagonistically control *AeSCP-2* transcriptional activity in the midgut of feeding larvae via the regulatory sequences between −1.6 to −1.3 kb 5′ upstream of the transcription start site. *In vivo* expression knockdown of *THAP* and *ATF-2* resulted in significant changes in developmental progression, which may be partially due to their effects on *AeSCP-2* expression.

## Introduction

Cholesterol is an important component of all animal cellular membranes and is the precursor for steroid hormone biosynthesis. Insects, unlike the vertebrates, are not able to synthesize cholesterol *de novo* due to the lack of key enzymes in the cholesterol synthesis pathway [1]. Therefore, insects must depend on dietary and/or symbiotic microbes to meet their physiological requirements for cholesterol [2], [3], [4], [5], [6]. Sterol carrier protein-2 (SCP-2) is a small intracellular protein involved in cholesterol and lipid intracellular trafficking in insects [7], [8]. AeSCP-2, the first insect SCP-2 identified from the yellow fever mosquito, *Aedes aegypti*, was found to bind to cholesterol [7] and fatty acids [9]. Moreover, functional studies of AeSCP-2 in *Aedes aegypti* revealed that AeSCP-2 plays an important role in cholesterol uptake, mosquito development and reproduction [10], [11].

It has been confirmed that the midgut and possibly foregut are the sites of cholesterol uptake and absorption in insects [12], [13], [14], [15]. In *Aedes aegypti*, AeSCP-2 was expressed strongly throughout feeding larval stages and decreased to low levels after pupation [7]. Interestingly, high levels of *AeSCP-2* expression are found in the larval midgut tissue [7]. Knockdown of AeSCP-2 gene expression *in vivo* effectively interferes with AeSCP-2 gene expression in the larval midgut, leading to the observed decreases in cholesterol uptake in larvae [10]. Results from earlier studies have indicated that AeSCP-2 gene expression is stage and tissue specific [7]. However, the transcriptional regulatory mechanism is unknown.

Results from the vertebrate SCP-2 gene transcriptional regulation studies have demonstrated that the vertebrate SCP-2 gene expression appears to be under the control of factors such as adrenocorticotropic hormone and gonadotropins via cyclic adenosine monophosphate (cAMP) activation [16]. In insects, 20-hydroxyecdysone (20E) up-regulates the transcription of *AeSCP-2* by 2-fold in cultured gut tissues [7], [17]. Promoter/reporter gene transfection assays in cultured mosquito Aag-2 cells demonstrated that 20E-induced up-regulation of *AeSCP-2* transcription requires HR3, an ecdysone-inducible transcription factor; and Ftz-F1, a 20E-responsive-late gene, may be involved in the down regulation of the AeSCP-2 gene [17]. There are significant increases in ecdysteroid levels in 24 hour-old larvae [18], [19], [20], which is sufficient to induce the expression of some 20E-inducible genes such as *HR3* and *E75*, but, not *Ftz-F1* [19]. Therefore, it is unlikely that Ftz-F1 is involved in *AeSCP-2* transcriptional regulation in Day 1 4^th^ instar larvae. Factors that control the temporal/spatial *AeSCP-2* expression *in vivo* in feeding larvae are unknown. Considering its critical role in mosquito’s cholesterol metabolism and development [10], [11], it is important to further investigate the mechanism of AeSCP-2 gene expression regulation. Based on the newly developed gene delivery method in *Aedes aegypti* [11], the *AeSCP-*2 promoter regulatory sequence and potential promoter regulatory proteins were studied *in vivo* in this study.

## Results

### Identification of the Spatial/temporal Regulatory Sequences in *AeSCP-2* Promoter

*AeSCP-2* expression during the 4^th^ instar is 5-fold higher in 24 hour-old larvae (feeding) than that of post feeding cessation at 62 hours post 3^rd^ molt [21]. In order to identify the temporal/spatial expression regulatory sequence in *AeSCP-2* promoter, six serial truncated promoter constructs [17] containing the CAT reporter gene were mixed with polyethylenimine (PEI) and microinjected to blood fed female mosquitoes at 16-18 hours the post blood meal (PBM). The CAT expression from the promoter/CAT constructs were measured in F0 4^th^ instar larval samples. The -0.06 kb construct showed significantly higher CAT expression levels than that of in the non-transfected larvae (non-transfected control vs. −0.06 kb, *p*<0.05, data not shown), suggesting that the −0.06 kb 5′ flanking sequence may contain the basal promoter that drove the basal level reporter gene expression.

**Figure 1:**
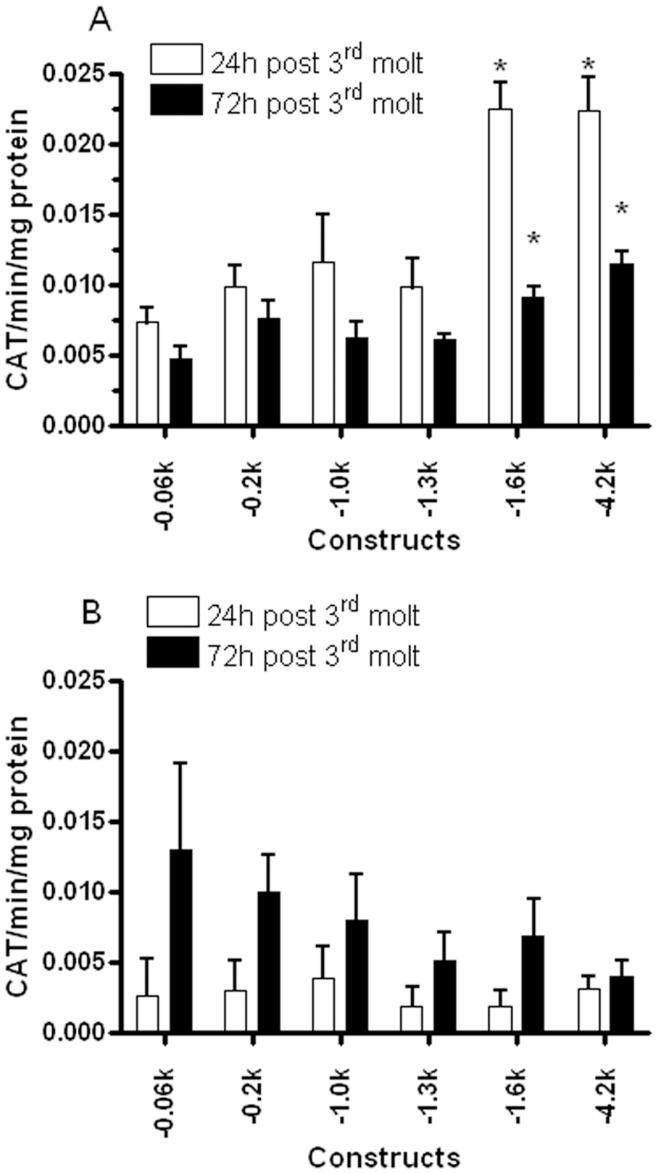
Quantitative analysis of *in vivo* CAT expression from promoter/CAT constructs. (**A**) The midgut samples from synchronized 4^th^ instar larvae (30 larvae/sample) were taken at 24 and 72 hours post 3^rd^ molt. (**B**) The carcass samples were from the same larvae as the midgut sampling. CAT quantity in each sample was defined as CAT unit/min of the reaction/µg protein. Mean and standard deviation are shown (N = 5−6). * Indicating significantly different (p<0.05) from the −0.06 kb construct at the same developmental time point.

In the larval midgut, there were no significant differences in levels of CAT expression driven by the −0.06, −0.2, −1.0, and −1.3 kb 5′ flanking sequence at both 24 hour-old (24 h) and 72 hour-old (72 h) 4^th^ instar larval stages (Fig. 1A, −0.06 to −1.3 kb), suggesting that temporal regulatory elements are not located within the −1.3 kb 5′ flanking sequence. The −4.2 kb and −1.6 kb 5′ flanking sequences showed 3.1- to 3.0-fold significantly higher CAT reporter gene expression (*p*<0.05) than that of the −0.06 kb construct in the midgut at 24 h time point, respectively(Fig. 1A). There was significantly (*p*<0.002) lower promoter activities driven by −1.3 kb than that of −1.6 kb in 24 hour-old samples (Fig. 1A), indicating that regulatory sequences between −1.6 and −1.3 kb is important for sustaining the high level of promoter activity in the feeding 4^th^ instar larvae. In the larval midgut, the promoter activities of the −4.2 and −1.6 kb constructs at 72 h decreased by 59% and 49% compared to 24 h, respectively (*p*<0.01, Fig. 1A). The results suggest that sequence upstream of −1.3 kb is important for temporal promoter activity in the midgut in feeding 4^th^ instar larvae. At the 72 h time point (feeding ceased), promoter activities of −4.2 and −1.6 kb were significantly lower than that of at 24 h, but, the levels were still significantly higher (*p*<0.05) than that of −0.06 kb (Fig. 1A), indicating that regulatory sequences upstream of −1.3 kb are responsible for the continued above basal level transcriptional activity of *AeSCP-2* in the midgut.

In the larval carcass samples, there were no significant differences in levels of CAT expression among the six truncated promoter constructs at 24 h time point (Fig. 1B). The results were in sharp contrast to increased promoter activities of −4.2 and −1.6 kb in the midgut at 24 h (Fig. 1A vs. B, 24 h), indicating that regulatory sequences upstream of −1.3 kb are important for the spatial promoter activity of *AeSCP-2*. Promoter activities of −0.06 kb was significantly increased at 72 h compared to 24 h (*p* = 0.043, Fig. 1B), whereas upstream −0.2 kb promoter activities did not change significantly in the carcass tissues overtime (Fig. 1B).

**Figure 2:**
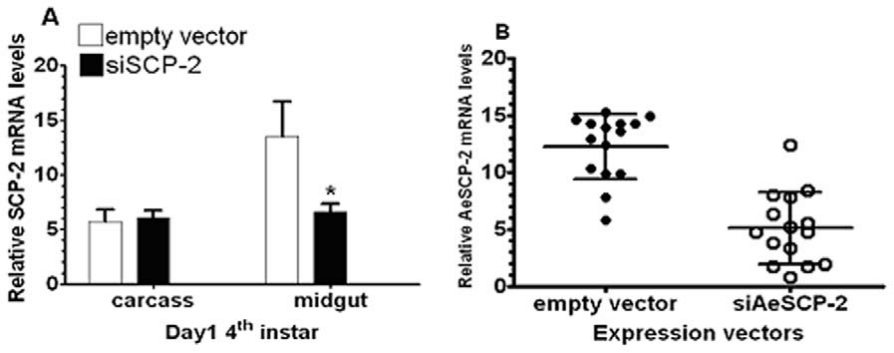
Tissue-specific transcriptional activities of the −1.6 kb *AeSCP-2* construct and efficiency for driving siRNA expression in transfected larvae. (**A**) Expression knockdown of *AeSCP-2* via the −1.6 kb *AeSCP-2* promoter driven siRNA expression. Tissues were collected from the same larvae (10 larvae/sample). Mean and standard deviation are shown (N = 3). (**B**) Fifteen synchronized 24 hour-old 4^th^ instar larvae were randomly selected and the total RNA was extracted from each individual larva. Mean and standard deviation are shown (N = 15). Relative (vs. *Actin-2*) AeSCP-2 mRNA levels from each sample were determined via RT-qPCR. * Indicating significant difference (p<0.05) from that of the empty vector construct.

To confirm that the −1.6 kb *AeSCP-2* promoter sequence would be sufficient to regulate tissue specific transcriptional activities, a siAeSCP-2 expression vector under the control of the −1.6 kb *AeSCP-2* promoter was constructed. Larvae transfected with the −1.6 kb-siAeSCP-2 construct via DNA/PEI injection of vitellogenic females were reared to 4^th^ instar. Larval tissues from synchronized 4^th^ instar F0 larvae were collected. To estimate the efficiency of delivery −1.6 kb-siAeSCP-2 construct into F0 larvae, RNA sample was also extracted individually from 15 randomly selected 24 h larvae from one batch of the transfection. Relative *AeSCP-2* mRNA levels (vs. *Actin-1*) were determined via RT-qPCR analysis. The −1.6 kb 5′ flanking sequence drove siAeSCP-2 expression that knocked down *AeSCP-2* expression by 51% in the midgut, whereas the level of *AeSCP-2* mRNA in the carcass was unchanged (Fig. 2A). The efficiency of the −1.6 kb AeSCP-2 promoter driven siRNA was 67% in randomly selected larvae that showed significantly reduced the targeted mRNA levels below the 50% average of the vector control (*p* = 0.02, Fig. 2B), which is consistent with previously reported *in vivo* transfection efficiency [11]. The lack of −1.6 kb driven siRNA expression knockdown in carcasses would not have been due to the lack of transfection in the larval carcass because heat shock inducible β-gal activities have been detected in 4^th^ instar larval carcass in previous studies using the same DNA/PEI delivery method [11]. The results of −1.6 kb/siRNA targeted tissue specific expression knockdown (Fig. 2A) is consistent with the −1.6 kb/CAT reporter gene assays in 24 h 4^th^ instar larvae (Fig. 1, −1.6 kb/CAT at 24 h, midgut vs. carcass ). The −1.6 kb 5′ flanking sequence of *AeSCP-2* is the first confirmed larval midgut specific promoter in *A. aegypti*.

### Potential Transcription Factors Bound to *AeSCP-2* Promoter Regulatory Region

Based on the temporal/spatial regulatory activities of the sequences between −1.6 to −1.3 kb 5′ upstream of *AeSCP-2* transcription start site (Fig. 1A and B), we focused on the 305 bp regulatory sequence between −1.6 to −1.3 kb 5′ flanking region of *AeSCP-2* in search for potential regulatory proteins. Biotin-labeled 305 bp DNA fragment of the −1.6 to −1.3 kb 5′ upstream sequence of *AeSCP-2* was amplified via PCR and used as probes for the biotin-streptavidin pull-down assay. Due to the promoter activities varied significantly in a temporal fashion between 24 hour-old and 72 hour-old 4^th^ instar larval midgut samples (Fig. 1A); midgut nuclear extracts from these two stages were used to search for the potential binding proteins that interact with −1.6 kb/−1.3 kb sequence of the promoter. Following the DNA probe/protein pull-down experiment, protein mixtures that bound to the labeled DNA probe were purified and the proteins were identified via LC-MS spectrometry (see M&M). There were six proteins bound to the 306 bp regulatory sequence in the midgut nuclear extract of 24 hour-old 4^th^ instar and six bound proteins were identified in the midgut nuclear extract of 72 hour-old 4^th^ instar (Table 1). AAEL002827, the ATP synthase beta subunit, was one common protein bound to the AeSCP-2 −1.6/−1.3 kb promoter sequence in both 24 h and 72 h larval midgut nuclear extracts (Table 1). We considered AAEL002827 as a non-specific contaminant in the pull-down assays.

**Table 1:** Nuclear proteins bound to the *AeSCP-2* −1.6/−1.3 kb regulatory sequence.

24 h 4^th^ instar larval stage	72 h 4^th^ instar larval stage
AAEL002827 (novel gene)ATP synthase beta subunit	AAEL002827 (novel gene)ATP synthase beta subunit
AAEL010577 (novel gene)Unknown protein containing THAP domain, homology of human THAP3	AAEL000494 (novel gene)histone H2A
AAEL011794 (novel gene)Unknown protein containing IBR domain	AAEL015674 (novel gene)histone H2B
AAEL013261 (VectorBase gene: AAEL801171)Protein containing C2H2-type zinc fingers, homolog of *Drosophila* ATF-2 (CG30420)	AAEL011795 (novel gene)allergen, putative
AAEL005286 (novel gene)Unknown protein containing CCCH-type Zinc finger domain	AAEL006857 (novel gene)Unkwon protein containing RRM domain
AAEL017566 (novel gene)Homolog of *Drosophila* DNA ligase 1 (CG5602)	AAEL001950 (novel gene)Unknown protein containing UBA domain

Of the five unique proteins bound to the −1.6/−1.3 kb probe in the 24 hour-old 4^th^ instar larval midgut nuclear extract, AAEL017566, is the homolog of *Drosophila* DNA ligase 1 (Table 1, 24 h) that is involved in DNA replication, repair, and recombination [22]. DNA ligase 1 is known to bind oligonucleotides [22], and it is likely that the labeled DNA probe functioned as an oligonucleotide substrate to pull DNA ligase 1 down. In the 24 h 4^th^ instar larval midgut nuclear extract, four of the −1.6/−1.3 kb pulled-down proteins are zinc finger transcription factors with unknown function in mosquitoes (Table 1, 24 h). Analysis of putative functional domains of these proteins revealed that the hypothetical protein AAEL010577 contains a Thanatos-associated protein (THAP) domain (DNA-binding domain) that is homologous to the human THAP3 and *Drosophila* CG14965 with 26.86% and 26.13% identity (54.23% and 50.81% similarity), respectively. Proteins containing THAP domain are involved in diverse biological processes [23]. AAEL013261 is the homolog of *Drosophila* activating transcription factor-2 (ATF-2) with 30.33% identity (63.12% similarity). The *Drosophila* ATF-2 has been shown to be involved in lipid metabolism [24]. AAEL011794 and AAEL005286 have orthologs only in mosquito species based on similarity search in the Blastp database (Blast database, NCBI).

**Figure 3:**
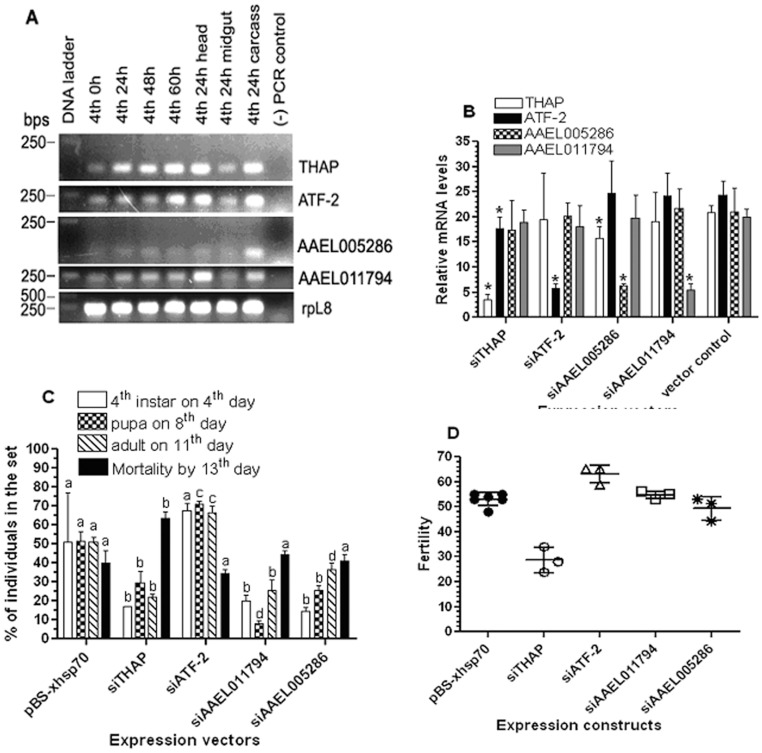
Effects of expression knockdown of the transcription factors (Table 1, 24 h) on the progression of development, mortality, and female fertility. The siRNA vector is driven by the *Drosophila hsp70* short promoter. Thirty larvae were synchronized on Day 1 2^nd^ instar, heat shock at 37°C started on Day 1 of 2^nd^ instar through pupal stage and adults were returned to 26°C. (**A**) Temporal/spatial transcription profiles of the transcription factors in 4^th^ instar larvae via semi-quantitative RT-PCR (30 cycles). (**B**) RNA sample of pooled 24 h 4^th^ instar larval midguts (10 larvae/sample) was taken from randomly selected larvae in each respective group (triplicate batches). Relative quantity of mRNA (vs. *rpL8*) was determined via RT-qPCR analysis. Mean ± standard deviation (N = 3). * Indicate significantly different (*p*<0.05, paired t-test) from the vector control. (**C**) Developmental progress and mortality was recorded daily. The same letter as the vector control above the bars represents that the mean values did not differ from the control significantly in paired t-test (*p*>0.05). Different letters above each construct represent that the mean values were significantly different between constructs (*p*<0.05). (**D**) Female fertility was measured as producing viable 2^nd^ instar larvae per blood-fed female (after the 1^st^ bloodmeal) in 3–6 batches (>5 female/batch) of each mating group. Each group in panel “A” had ≥5 surviving pairs per batch. Mean and standard deviation are shown (N = 3−6).

Endogenous expression of the transcription factors (Table 1, 24 h) in 4^th^ instar larvae were confirmed via semi-quantitative RT-PCR analysis. The mRNA levels of *THAP* were high between 24–60 h in the 4^th^ stadium and were much lower in the midgut than that of in the head and the carcass in 24 hour-old 4^th^ instar larvae (Fig. 3A, THAP). Increased *ATF-2* transcription was detected only in 60 hour-old 4^th^ instar larvae when feeding had ceased and the expression in the midgut was much lower than that of in other tissues in 24 hour-old 4^th^ instar larvae (Fig. 3A, ATF-2). The levels of *AAEL005286* and *AAEL011794* transcripts did not show detectable change throughout 4^th^ instar and the mRNA levels in the larval midgut were lower than that of in the other tissues (Fig. 3A). The results indicate that all four transcription factor genes were actively transcribed in 4^th^ instar larvae and detectable levels of transcripts were found in the 24 h 4^th^ instar larval midgut, where *AeSCP-2* expression is high [7]. Therefore, it is possible that those transcription factors may regulate *AeSCP-2* transcription in the larval midgut.

Those transcription factors (Table 1, 24 h) were expressed in the larval midgut at approximately similar levels (Fig. 3B, vector control). To determine whether those transcription factors have essential functions *in vivo*, expression knockdown via siRNA vectors was carried out for each of the transcription factor. Each perspective siRNA effectively knocked down its own gene expression by at least 70% in the 24 h 4^th^ instar larval midgut (Fig. 3B). To determine the effect of siRNA of each perspective transcription factor (Fig. 3B) on each other’s expression, mRNA levels of each transcription factor in the midgut was measured via RT-qPCR in samples of siRNA-treated larvae. Interestingly, siTHAP also reduced *ATF-2* expression by 27% (Fig. 3B, siTHAP and *ATF-2*), whereas siAAEL005286 led to a 24% decrease in *THAP* expression (Fig. 3B, siAAEL005286 and *THAP*). The expression knockdown effects of siTHAP and siAAEL005286 on *ATF-2* and *THAP* transcription were not due to the hairpin sequences of the perspective gene since there was no sequence similarity between the siTHAP and siAAEL005286 and *ATF-2* and *THAP* mRNA, respectively (see M&M).

Thirty F0 larvae transfected with a siRNA expression vector were synchronized on Day 1 2^nd^ instar and heat shocked at 37°C as described in M&M. Mortality and larval development was recorded daily, surviving adults were allowed to mate within each group and the female fertility was recorded after the 1^st^ bloodmeal on adult day 4^th^. Heat-shock at 37°C throughout the 2^nd^ -pupal stages resulted in a high mortality rate at 40% in control groups (Fig. 3C, total death by 13^th^ day, pBS-xhsp70). However, siTHAP groups had significantly higher mortality rate (a 23% increase) than that of in the vector control (Fig. 3C, pBS-xhsp70 vs. siTHAP, *p* = 0.0004, *t* = 6.944 *df* = 6). Most of the increased mortality in the siTHAP group occurred in the pupal stage. On the other hand, the mortality rates in siATF-2, siAAEL011794, and siAAEL005286 groups were similar to the vector control (Fig. 3C, pBS-xhsp70 vs. siATF-2, siAAEL011794, and siAAEL005286). The results suggest that heat shock alone was not the only cause of higher mortality in siTHAP-treated groups. It is likely that THAP plays some roles in survivorship. *In vivo* expression knockdown of *THAP*, *AAEL011794*, and *AAEL005286* significantly delayed larval development, pupation and adult emergence compared to the vector control (Fig. 3C, pBS-xhsp70 vs. siTHAP, siAAEL011794, and siAAEL005286, *F*_1,21_ = 28.02, 39.21, and 23.34, respectively, *p*<0.0001). Interestingly, siATF-2-treatment led to significantly accelerated developmental pace compared to the vector control (Fig. 3C, pBS-xhsp-70 vs. siATF-2, *F*_1.21_ = 10.76, *p* = 0.0036). Female fertility in the vector control groups (Fig. 3B) was similar to previously reported [11]. There was a 52% decrease in fertility in siTHAP-treated groups compared to the control (Fig. 3D, pBS-xhsp70 vs. siTHAP, *p* = 0.0158, *t* = 7.863 *df* = 2), whereas there was a 22% increase in fertility in siATF-2-treated groups (Fig. 3D, pBS-xhsp70 vs. siATF-2, *p* = 0.0216, *t* = 4.414 *df* = 3). Treatments with siAAEL011794 or siAAEL005286 did not significantly alter the female fertility compared to the control group (Fig. 3D, pBS-xhsp70 vs. siAAEL011794 and siAAEL005286). The results showed that all four transcription factors may be involved in the control of development progression in *Aedes aegypti*, but only THAP and ATF-2 were likely contributing to female fertility.

In the 72 h 4^th^ instar larval midgut nuclear extract, two histone proteins, the histone H2A and H2B, was found to bind to the −1.6/−1.3 kb regulatory sequence (Table 1, 72 h). Both histone H2A and H2B are in the core structure of nucleosome that involves in chromatin remodeling machinery [25], suggesting that the down-regulation of *AeSCP-2* expression in the larval midgut post larval feeding stage might be due to chromosome condensation. Three other proteins from the 72 h nuclear extract, AAEL006857, AAEL001950, and AAEL011795, have no DNA-binding motifs (Table 1, 72 h) and are functionally unknown proteins in mosquitoes. AAEL001950 has an Ubiquitin Associated domain (UBA) close to the C-terminal and an Ubiquitin-like domain close to the N-terminal. AAEL011795 has a CAP domain that is found mostly in extracellular proteins [26]. AAEL006857 has a RNA recognition motif (RRM) that is involved in RNA and protein interactions [27], [28].

### *In vivo* Regulatory Functional Study of Transcription Factors on AeSCP-2 Expression

**Figure 4:**
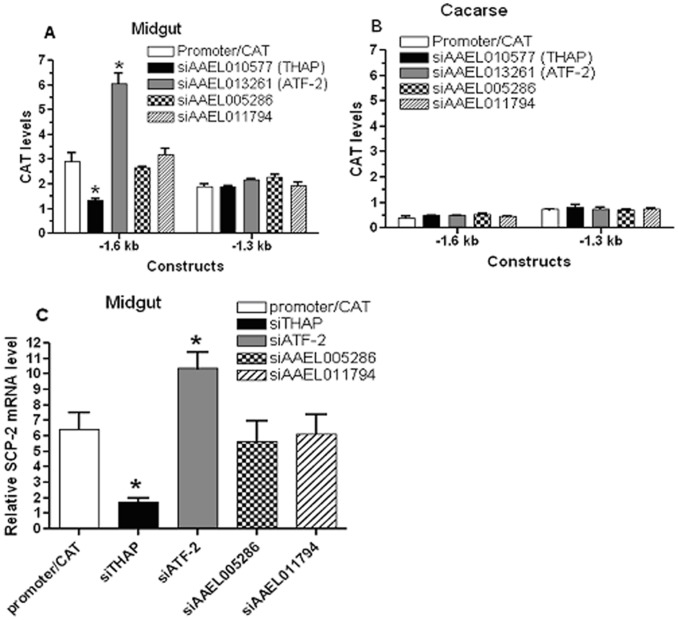
Quantitative analysis of *in vivo* CAT expression from co-transfection of transcription factor siRNA vector and AeSCP-2 promoter/CAT constructs (1∶1 ratio). In the promoter/CAT group, *hsp70* short promoter vector plasmid was added in at 1∶1 ratio to normalize the total amount of promoter/CAT each group received. Larvae were synchronized on Day 1 2^nd^ instar, heat shocked at 37°C for 24 hours on Day 1 of 2^nd^ and 4^th^ instar, respectively. Day 1 4^th^ instar larval samples were taken after the 2^nd^ heat shock-treatment. (**A**) The midgut samples from 4^th^ instar larvae (30 larvae/sample) were taken at 24 hour post 3^rd^ molt. (**B**) The carcass samples were from the same larvae as the midgut sampling. (**C**) Larval midgut endogenous transcription of *AeSCP-2* in −1.3 kb/CAT and siRNA co-transfected larvae. Ten synchronized 24 h 4^th^ instar larvae were pooled from each batch of the co-transfection experiment (in panel A, −1.3 kb). Relative *AeSCP-2* mRNA levels (vs. *Actin-1*) from each sample were determined via RT-qPCR. Mean and standard deviation are shown (N = 3). * Indicating significantly different (*p*<0.05) from the promoter/CAT control.

To confirm that the transcription factors identified above control *AeSCP-2* expression via the −1.6/−1.3 kb promoter sequence, we performed *in vivo* promoter/reporter gene assays under the condition of expression knockdown of each transcription factor. F0 larvae were hatched and synchronized on Day 1 2^nd^ instar. Selected Day 1 2^nd^ instar larvae were heat shocked for 24 hours at 37°C and returned to 26°C, a second heat shock at 37°C for 24 hours was given to Day 1 4^th^ instar to ensure continuous high levels of expression knockdown throughout the life cycle (Fig. S2). Quantities of CAT reporter gene product in 24 h 4^th^ instar larval midgut and carcass were determined. The levels of expression knockdown of each transcription factor were determined via RT-qPCR analysis for triplicates of 10 pooled larvae from the same batch for each of the promoter/reporter gene assays (Fig. 3B). Three experimental repetitions were performed for each promoter/reporter gene construct. In the promoter/CAT control groups, the −1.6 kb construct drove significantly higher levels of reporter gene expression than that of −1.3 kb construct in 24 h 4^th^ instar larvae (*p* = 0.047, Fig. 4A), which is consistent with the results in figure 1A (Fig. 1A, −1.3 kb vs. −1.6 kb in 24 h samples). The absolute CAT levels in each corresponding tissue sample were lowered in the reporter gene construct/siRNA co-transfected larvae than that of the reporter gene construct alone (Fig. 1 vs. Fig 4), this might be due to the amount of reporter gene constructs injected in the co-transfection experiments being only ½ of the amount of reporter gene construct alone assays (see M&M).

Under the condition of expression knockdown of *THAP*, the −1.6 kb/CAT transcriptional activities in the midgut decreased to 45% of the control (*p*<0.05, Fig. 4A), whereas under the same conditions there was no significant change in reporter gene expression in the carcass samples (Fig. 4B). On the other hand, siTHAP expression did not affect the −1.3 kb promoter activity in the midgut (Fig. 4A). The results suggest that THAP may be required for maintaining high levels of *AeSCP-2* transcription in the 24 h 4^th^ instar larval midgut. It is highly likely that the THAP regulatory element resides in the −1.6/−1.3 kb 5′ flanking sequence. Under the condition of *ATF-2* expression knockdown, a 2-fold increase in −1.6 kb transcriptional activities was detected only in the midgut compared to the vector plasmid control (*p*<0.05, Fig. 4A and B). Expression knockdown of *ATF-2* had no effect on the promoter activity of −1.3 kb in either the midgut or the carcass tissues (Fig. 4A and B). The results suggest that ATF-2 may attenuate *AeSCP-2* expression in the larval midgut via the regulatory sequence in the −1.6/−1.3 kb region. However, neither *AAEL005286* nor *AAEL011794* expression knockdown had significant effects on the *in vivo* −1.6/−1.3 kb transcriptional activity in 24 h 4^th^ instar larvae (Fig. 4A and B). The results showed that THAP and ATF-2 antagonistically regulated *AeSCP-2* promoter activities via the −1.6/−1.3 kb regulatory sequence.

To verify the quantitative effect of expression knockdown of the transcription factors on the endogenous *AeSCP-2* expression *in vivo*, we took larval midgut RNA samples from 24 h 4^th^ instar larvae (10 larvae/sample) in the −1.3 kb/CAT and siRNA co-transfection batches and measured the endogenous *AeSCP-2* mRNA levels via RT-qPCR analysis. *AeSCP-2* mRNA levels in the midgut decreased by 73.2% in siTHAP-treated larvae (*p*<0.05, Fig. 4C, siTHAP vs. promoter/CAT). When *ATF-2* expression was knocked down by siATF-2, the *AeSCP-2* mRNA level in the 4^th^ instar larval midgut increased to 161% of the control (*p*<0.05, Fig. 4C, siATP vs. promoter/CAT). Expression knockdown of *AAEL005286* or *AAEL011794* had no effect on *AeSCP-2* transcription in the larval midgut (Fig. 4C). The effects of transcription factor siRNA treatment on *AeSCP-2* expression in larvae showed that THAP and ATF-2 played a regulatory role in the endogenous *AeSCP-2* transcription.

### Effects of Expression Knockdown of *THAP* and *ATF-2* on Development and Growth

It has been shown that *in vivo* expression knockdown of *AeSCP-2* results in delayed development, higher mortality, and lower female fertility [10], [11]. Since THAP is implicated in maintaining high levels of *AeSCP-2* expression in feeding larvae *in vivo* (Fig. 4), we expected that the lowered *AeSCP-2* expression via siTHAP treatment would result in similar phenotypic responses as that of siAeSCP-2 treatment. We speculated that ATF-2 is an *AeSCP-2* suppressor; therefore, increased *AeSCP-2* expression via siATF-2 treatment would rescue siAeSCP-2-induced effects on development, growth and fertility. It is likely that both THAP and ATF-2 have more than one targeted gene, however, manipulation of *in vivo AeSCP-2* expression via siRNA or over-expression vectors may shed light on the functional role of THAP and ATF-2 in *AeSCP-2* transcriptional regulation that impacts growth and development. The constitutive AeSCP-2EGFP fusion protein expression vector [29] was co-transfected with siTHAP to “rescue” the negative effect of *THAP* expression knockdown on *AeSCP-2* expression (Fig. S1, AeSCP-2, lanes 2 and 3 vs. 4 and 5). The AeSCP-2EGFP fusion protein shows similar cellular function as that of AeSCP-2 in cultured mosquito cells [29] and is easily distinguishable from the wild type AeSCP-2 via western blotting analysis (Fig. S1, AeSCP-2EGFP, lanes 2–4). The siSCP-2 vector was co-transfected with siATF-2 to overcome the positive effect of *ATF-2* expression knockdown on *AeSCP-2* transcription.

**Figure 5:**
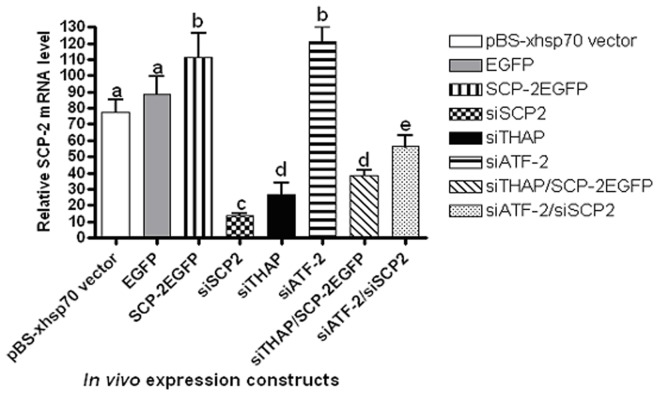
*In vivo* AeSCP-2 transcription in the larval midgut under the influence of transfected expression vectors. The siRNA vector is driven by the *Drosophila hsp70* short promoter. Constitutive over expression is drive by the Baculovirus immediate early gene promoter [17]. In the co-transfected groups, two expression vectors were added at 1∶1 ratio. In the single expression vector groups, pBS vector plasmid was added in at 1∶1 ratio to normalized the total amount of expression vector each group received. Larvae were synchronized on Day 1 2^nd^ instar; siRNA expression was induced via heat shocked at 37°C for 24 hours on Day 1 of 2^nd^ and 4^th^ instar, respectively. Heat shock-treatment was not applied to the EGFP and SCP-2EGFP groups. Day 1 4^th^ instar larvae (10 larval midguts/sample) were taken after the 2^nd^ heat shock-treatment. Relative (vs. *rpL8*) AeSCP-2 mRNA levels from each sample were determined via RT-qPCR. Mean and standard deviation are shown (N = 3). The same letters above the bars in each construct represent that the mean values did not differ from other constructs significantly (p>0.05) in paired t-tests with the vector control.

Transfected larvae were synchronized on Day 1 2^nd^ instar and heat shocked at 37°C for 24 hours on Day 1 of 2^nd^ and 4^th^ instar, respectively. Midgut samples were taken from 24 h 4^th^ instar larvae. There was no significant difference in *AeSCP-2* expression in larvae transfected with either the siRNA expression vector or over-expression vector (Fig. 5, pBS-xhsp70 vector vs. EGFP). In siSCP-2-treated larvae, *AeSCP-2* mRNA level decreased by 82.5% (*p*<0.05, Fig. 5), showing that the *in vivo* heat shock-induced siRNA expression functioned well. Expression knockdown of *THAP* resulted in a 65.3% decline in *AeSCP-2* transcript (*p*<0.05, Fig. 5), which were consistent with the endogenous *AeSCP-2* expression in larvae from the promoter/CAT reporter in assays (Fig. 4C, siTHAP). It is noted that regardless which internal control genes were used in RT-qPCR analyses (Fig. 4C using *Actin-1* as the internal control vs. Fig. 5 using *rpL8* as the internal control) there were no significant differences in the percentage of changes in levels of *AeSCP-2* expression in siTHAP- and siATF-2-treated groups vs. vector controls, i.e. the % changes in *SCP-2* mRNA levels in figure. 4C compared to figure 5 (*p* = 0.25 and 0.66 for siTHAP and siATF-2, respectively). Over-expression of AeSCP-2EGFP driven by the Baculovirus immediate early gene promoter (IE [17]) did not significantly increase *AeSCP-2* mRNA levels in the larval midgut (Fig. 5, EGFP vs. SCP-2EGFP, *p* = 0.10), which did not significantly rescued the negative effect of siTHAP treatment on *AeSCP-2* transcription compared to the pBS-xhsp70 vector control (Fig. 5, siTHAP vs. siTHAP/SCP-2EGFP, *p* = 0.07). The results indicate that the Baculovirus immediate early gene promoter was not sufficiently active in the larval midgut to drive high levels of target gene expression. On the other hand, siAeSCP-2 expression was able to effectively damp the positive effect of siATF-2 on *AeSCP-2* transcription from 156% to 63.7% of the vector controls (Fig. 5, siATF-2 vs. siATF-2/SCP-2EGFP, *p*<0.05). The results showed that varied levels of *in vivo AeSCP-2* expression were achieved effectively in most cases via combination of expression vectors.

**Figure 6:**
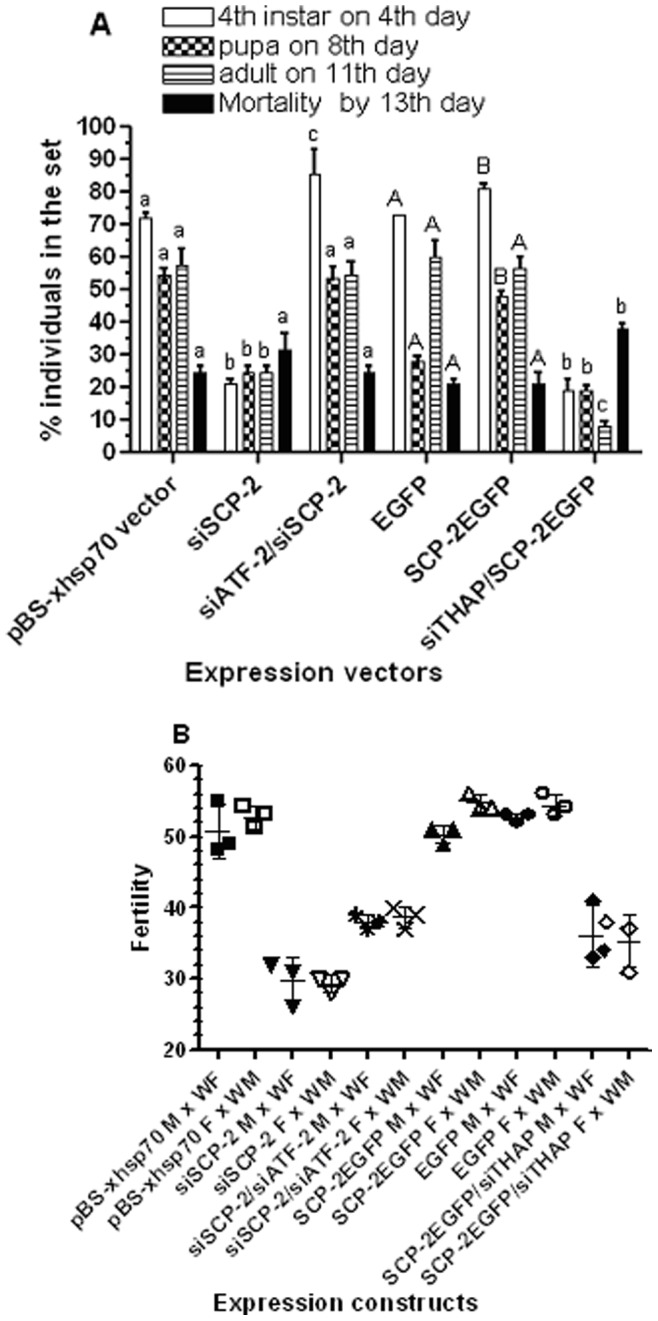
Effects of *THAP* and *ATF-2* expression knockdown on development and fertility. The siRNA vector is driven by the *Drosophila hsp70* short promoter (see M&M). Constitutive over expression is drive by the immediate early gene promoter (Vyazunova and Lan, 2010). In the co-transfected groups, two expression vectors were added at 1∶1 ratio. In the single expression vector groups, pBS plasmid was added in at 1∶1 ratio to normalized the total amount of expression vector each group received. Thirty larvae were synchronized on Day 1 2^nd^ instar; siRNA expression was induced via heat shocked at 37°C for 24 hours on Day 1 of 2^nd^ and 4^th^ instar, respectively. Heat shock-treatment was not applied to the EGFP and SCP-2EGFP groups. (**A**) Developmental progression and mortality. The same letters above the bars in each construct represent that the mean values did not differ from other constructs significantly (p>0.05) in paired t-tests within each observation. Lower case letter represents heat shock treated group, capital letter represents non-heat shocked group. (**B**) Female Fertility (after the 1^st^ bloodmeal). Fertility is defined as viable 2^nd^ instar larvae/female. Survived pupae in each group from (Fig. 6A) were separated by sex and adults emerged in separated cages. Adults from each group (8–12) were mated with 10 wild type opposite sex (WF = wild type female or WM = wild type male). Eggs of blood-fed females were hatch 5–6 days after egg deposition. The average fertility/female in each mating group is presented. Mean and standard deviation are shown (N = 3). The same letters above the bars in each construct represent that the mean values did not differ from other constructs significantly (p>0.05) in paired t-tests within each observation. Capital letters above the bar represent non-heat shock groups; lower case letters above the bar represent heat shock-treated groups.

Thirty larvae from each batch were synchronized on Day 1 2^nd^ instar and siRNA expression was induced via heat-shock at 37°C for 24 hours on Day 1 of 2^nd^ and 4^th^ instar, respectively. Larval growth and development were recorded daily until the 13^th^ day when the adults were 3–5 days post emergence. The time that took to reach developmental milestones such as molting to 4^th^ instar, pupation, and adult emergence in each batch of transfection was compared to the control (transfected with empty expression vector). For the batches that transfected only with over-expression vector, no heat shock treatment was applied. Heat shock on day 1 2^nd^ instar for 24 hours did not significantly alter the pace of development to 4^th^ instar (Fig. 6A, 4^th^ instar on 4^th^ day, pBS-xhsp70 vector vs. EGFP). After the second 24 hour-heat shock on day 1 4^th^ larvae in the heat shocked groups reached to pupation and adult emergence at an accelerated pace compared to non-heat shocked control (Fig. 6A, pupa on 8^th^ day and adults on 11^th^ day, pBS-xhsp70 vector vs. EGFP, *F*_1,12_ = 316.5, *p*<0.0001), although there was no significant difference in total mortality between groups of heat shock vs. non-heat shock controls (Fig. 6A, total death by 13^th^ day, pBS-xhsp70 vector vs. EGFP). Heat-shock treatments at 37°C induced siAeSCP-2 expression (Fig. 5, siSCP-2) and resulted in delayed development compared to the vector control (Fig. 6A, pBS-xhsp70 vector vs. siSCP-2, *F*_1,12_ = 884.1, *p*<0.0001), which was consistent with previous report in larvae with 3-hour 42°C heat shock-treatment (Peng et al., 2011). Co-transfection of siATF-2 with siSCP-2 vectors led to significant recovery of siSCP-2-induced developmental delay (Fig. 6A, siATF-2/siSCP-2 vs. siSCP-2, *F*_1,12_ = 912.1, *p*<0.0001). The results seemed to correlate to the rescued levels of *AeSCP-2* transcription mediated by siATF-2 treatment (Fig. 5, siSCP-2 vs. siATF-2/siSCP-2). Over-expression of AeSCP-2EGFP resulted in a fast developmental pace in treated larvae (Fig. 6A, EGFP vs. SCP-2EGFP, *F*_1,12_ = 514.3, *p*<0.0001), however, the moderate effect of SCP-2EGFP on *AeSCP-2* expression in siTHAP treated group (Fig. 5, siTHAP vs. siTHAP/SCP-2EGFP) did not overcome the siTHAP-induced developmental delay (Fig. 6A, SCP-2EGFP vs. SCP-2EGFP/siTHAP). In fact, the delay in development was more severe in siTHAP/SCP-2EGFP-treatment compared to siAeSCP-2 alone (Fig. 6A, siSCP-2 vs. siTHAP/SCP-2EGFP, *F*_1,12_ = 8.333, *p* = 0.0137), suggesting that THAP may have additional target gene(s) that impact growth and development.

Heat shock- or osmotic stress-induced phosphorylation of dATF-2 results in its release from heterochromatin [30], which leads to changes in target gene expression [30], [31]. We used heat shock-treatment to induce siATF-2 expression; therefore, it is possible that heat-shock might activate the transcriptional function of endogenous ATF-2, which may in turn lead to down-regulation of *AeSCP-2* expression. However, we did not detect heat-shock-mediated changes in *AeSCP-2* expression (Fig. 5, pBS-xhsp70 vector vs. EGFP), indicating that heat shock alone (presumable stress-induced activation of endogenous ATF-2) was not sufficient to regulate *AeSCP-2* transcription. We showed that it was the heat-shock-induced siATF-2 expression that led to the up-regulation of *AeSCP-2* transcription in the midgut via the −1.6/−1.3 kb 5′ flanking sequence (Fig. 4A and C). Moreover, the siATF-2-induced accelerated growth and development (Fig. 2A, pBS-xhsp70 vs. siATF-2) was significantly reduced by the co-expression of siAeSCP-2 (Fig. 6A, pBSxhsp70 vector vs. siSCP-2/siATF-2). It is possible that expression knockdown of *ATF-2* under the condition of heat-shock exacerbated the effect of ATF-2 on *AeSCP-2* regulation; the results did suggest that ATF-2 is involved in *AeSCP-2* expression regulation.

Males and female adults from above batches were mated with wild type (non-transfected) and eggs/blood-fed female (the average of eggs/female in 3 batches of 5–7 females/sample), % of egg hatching, and number of surviving 2^nd^ instar from each mating group were recorded. Female fertility was defined as viable 2^nd^ instar/blood-fed female produced in each mating group. It should be noted that the data from egg/female were not the life time egg production of the female; instead, they were only the egg production after the 1^st^ bloodmeal. We previously reported that siAeSCP-2-treated pairs have significantly lower fertility than that of the control pairs [11]. However, the backcross to non-transfected sexes separately also had significantly lowered fertility compared to controls (Fig. 6B, pBS-xhsp70 M × WF vs. siSCP-2 M × WF and pBS-xhsp70 F × WM vs. siSCP-2 F × WM), the egg hatching rate in “siSCP-2 M × WF” and “siSCP-2 F × WM” were similar to reported “siSCP-2 pairs” [11]. The results suggest that the function of AeSCP-2 in males affected mated female’s fertility by unknown mechanisms. Co-transfection of siATF-2 with siSCP-2 led to significantly recovery of fertility (31% increase) in the backcross of both sexes compared to siSCP-2-treatment alone (Fig. 6B, siSCP-2/siATF-2 vs siSCP-2, *p*<0.05), which seemed to correlate to the significantly siATF-2-rescued *AeSCP-2* expression in the co-transfected larvae (Fig. 5, siATF-2/siSCP-2 vs. siSCP-2). Over-expression of SCP-2EGFP did not affect the fertility compared to controls (Fig. 6B, EGFP vs. SCP-2EGFP). However, co-transfection of SCP-2EGFP with siTHAP did not significantly affect siTHAP-mediated reduced fertility (Fig. 6B, SCP-2EGFP vs. SCP-2EGFP/siTHAP). The results suggest that either over-expression of SCP-2EGFP was not sufficient to overcome the siTHAP-mediated down-regulation of endogenous *AeSCP-2* expression (Fig. 5, SCP-2EGFP vs. SCP-2EGFP/siTHAP) or THAP targets more genes than just *AeSCP-2* that affect fertility.

It is unlikely that AeSCP-2 gene is the only target of THAP and ATF-2 based on the effects of siTHAP and siATF-2 on development progression and fertility. The siTHAP-treatment was significantly less effective in knocking down *AeSCP-2* expression than that of siAeSCP-2 (Fig. 5), although siTHAP/SCP-2EGFP-treated larvae experienced a significantly more severe delayed developmental progression (Fig. 6A, siSCP-2 vs. siTHAP/SCP-2EGFP, *F*_1,12_ = 8.333, *p* = 0.0137), noting that there were no significant differences in *AeSCP-2* transcription between siTHAP and siTHAP/SCP-2EGFP treatments (Fig. 5). The results suggest that THAP may control *AeSCP-2* and other gene(s) involved in growth and development. Over-expression of SCP-2EGFP resulted similar levels of *AeSCP-2* transcripts as that of in siATF-2-treated larvae (Fig. 5, siATF-2 vs. SCP-2EGFP). However, over-expression of AeSCP-2EGFP did not lead to higher female fertility (Fig. 6B, SCP-2EGFPxW vs. EGFPxW) as that observed in siATF-2-treatment (Fig. 3D, siATF-2 vs. pBS-xhsp70 vector), implying that ATF-2 targets other genes involving in fertility. It is noted that THAP and AFT-2 expression are not tissue-specific and the higher levels of mRNA was detected in the carcass (Fig. 3A), therefore, expression knockdown in the whole body including the midgut (Fig. 3B) may lead to biological effects beyond the midgut physiology. The larval midgut specific target expression knockdown using the *AeSCP-2′*s −1.6 kb 5′ flanking sequence would enable us to test the *in vivo* function of THAP and ATF-2 in the larval midgut in future studies.

## Discussion

High levels of *AeSCP-2* expression are found in the larval midgut tissues [7] and *AeSCP-2* transcription maintains at high levels throughout the feeding stage and decrease significantly 60 hours post 3^rd^ molt in developing pharate pupae [21]. Results from earlier *in vitro* studies have indicated that 20E/HR3 up-regulates the −1.0 kb *AeSCP-2* promoter activity, whereas 20E/Ftz-F1 down-regulates −1.6 kb *AeSCP-2* promoter activity in cultured mosquito cells [17]. Using the newly developed extrachromosomal *in vivo* expression method in *Aedes aegypti* [11], we evaluated the *in vivo AeSCP-2* promoter activities of six truncation constructs in 4^th^ instar larvae. A regulatory element between −1.6 and −1.3 kb 5′ flanking sequence interacts with in an endogenous factor in culture mosquito cells and that 20E/Ftz-F1 significantly weakens this interaction [17]. However, there is no detectable level of *Ftz-F1* expression in Day 1 4^th^ instar larvae [19]. Therefore, it is unlikely that Ftz-F1 is involved in *AeSCP-2* transcriptional regulation in Day 1 4^th^ instar larvae. Factors that control the temporal/spatial *AeSCP-2* expression *in vivo* in feeding 4^th^ instar larvae are unknown. To search for regulatory factors that control *AeSCP-2* transcription *in vivo*, it is necessary to define the 5′ flanking region that determines the temporal/spatial expression pattern. Using microinjection of promoter/reporter gene constructs to define temporal/spatial specific regulatory sequence *in vivo* has only been reported in *Aedes aegypti* adults [32], to study promoter activities in mosquito larvae via direct promoter/reporter DNA vector microinjection would be impossible due to high mortality rate post microinjection in 4^th^ larvae [10]. Using the newly developed extrachromosomal *in vivo* expression method in *Aedes aegypti* [11], we evaluated the *in vivo AeSCP-2* promoter activities of six truncation constructs in 4^th^ instar larvae.

To investigate transcription factors that regulate high levels of AeSCP-2 expression in the larval midgut during feeding, we focused our attention to the transcription factors identified in the 24 h sample. THAP and ATF-2 were found to antagonistically regulate the AeSCP-2 promoter activities via the −1.6/−1.3 kb 5′ flanking sequence in the larval midgut (Fig. 4A and B). THAP proteins are known to bind to DNA regulatory elements [33], [34] as well as interact with other proteins, both THAP/DNA and THAP/protein interactions lead to changes in the target gene expression [35], [36]. Similarly, ATF-2 is known to bind to regulatory sequence as well as to interaction with other regulatory proteins in vertebrates [37]. Whether AAEL011794 and AAEL005286 were pulled down by the −1.6/−1.3 kb regulatory sequence of *AeSCP-2* through the interaction with THAP and ATF-2 is unknown. AAEL011794- and AAEL005286-controlled development progression (Fig. 3C, pBS-xhsp70 vs. siAAEL011794 and siAAEL005286) is unlikely through the direct regulation *AeSCP-2* expression (Fig. 4), but through other unidentified target gene (s). Interestingly, the −1.6/−1.3 kb temporal/spatial regulatory sequence did not pull down any known 20E-regulated gene product (Table 1). There is no predicted ecdysone receptor regulatory element (EcRE) within the −1.6/−1.3 kb region [17]. The results suggest that 20E-regulated AeSCP-2 expression in the larval midgut [7], [17] may be via further upstream sequences or via indirect regulations. Whether ATF-2 and THAP expression is under the influence of 20E *in vivo* needs further investigation.

THAP belongs to a family of proteins with a N-terminal C2CH zinc finger DNA-binding domain [23] that shares similarity with the DNA-binding domain of *Drosophila P* element transposase [34], [38]. The mammalian THAP proteins show diverse function such as cell proliferation regulation [39], [40], cell cycle progression control [41], and transcription repression via inhibition of histone acetylation [35]. In *C. elegans*, there are five proteins containing the THAP domain that are involved in chromatin-modifying [42], larval growth [43], cell cycle regulation [44], and gene expression regulation via the recruitment of another transcription factor [45]. In insects, *P* element transposase contains an N-terminal THAP domain and is known to bind to the consensus THAP binding site [34]. However, functions of other *Drosophila* THAP domain proteins are unknown [23]. We showed that *Aedes* THAP is involved in maintaining high levels of *AeSCP-2* transcription in the larval midgut (Fig. 4). In the 5′ flanking sequence of AeSCP-2 gene (FJ554568) from −1573 to −1566 bps upstream of the transcription site, the 5′-TACGGGTA-3′ sequence matches 100% to the consensus THAP binding sequence of 5′-TXXGGGX(A/T)-3′ [34]. Whether AeTHAP binds to the −1.6/−1.3 kb AeSCP-2 5′ flanking sequence via the putative THAP binding site (5′-TACGGGTA-3′) needs further investigation.

ATF-2 is a C2H2 zinc finger transcription factor. ATF-2 has diverse functions depending on the developmental time and tissue. In mammalians, ATF-2 is important for normal cellular development and survival through its phosphorylation by JNK/p38 or ATM/ATR respectively [37], [46]. Mammalian ATF-2 is implicated in the control of CNS development in embryos [47]. It has been shown that *Drosophila* ATF-2 (dATF-2) positively regulates the transcription of *dPEPCK* (a key enzyme for both gluconeogenesis and glyceroneogenesis) via several CRE half-sites in the *PEPCK* promoter [24]. There is no sequence within 305 bps of the −1.6/−1.3 kb region in the *AeSCP-2* promoter (FJ554568) matching 100% to the ATF/CRE core binding site (5′-TGACGTCA-3′) of the ATF-2 responsive element. The ATF-2 responsive element in the −1.6/−1.3 kb region in the *AeSCP-2* promoter needs further investigation. SCP-2 is not a known ATF-2 target gene [24], [31]. This is the first report of ATF-2′s regulatory role in regulating an intracellular lipid carrier protein gene. In *Drosophila*, expression knockdown of *dATF-2* leads to smaller triglyceride reserves [24], [48] via dATF-2 regulated expression of PEPCK (dPEPCK) gene and decreased survival under starvation conditions [24]. We did not test siATF-2-treated larvae under starvation conditions. Therefore, whether AeATF-2 has similar *in vivo* function as that of dATF-2 is unknown.

**Figure 7:**
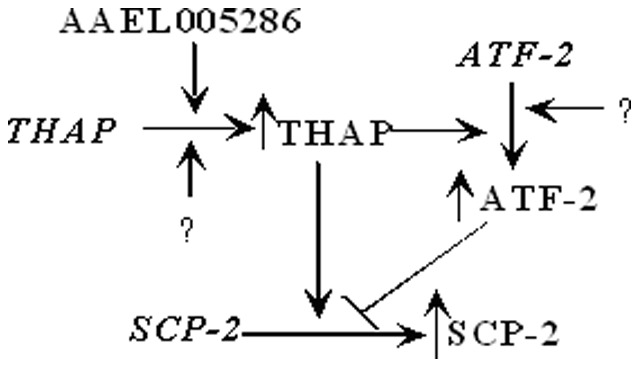
Schematic diagram showing THAP- and ATF-2-regulated AeSCP-2 expression in the midgut of feeding 4^th^ instar larvae. Arrows indicate up regulation, bar represents down regulation, “?” denotes unknown factors.

The antagonistic role of THAP and ATF-2 in *AeSCP-2* expression regulation in the larval midgut is intriguing. However, the regulation of *AeSCP-2* expression may be more complex considering that other factor(s) may control *AeSCP-2* expression indirectly. Expression knockdown of *AAEL005286* led to a 24% reduction in *THAP* expression that affected neither *ATF-2* nor *AeSCP-2* mRNA levels (Fig. 3B and Fig. 4C), although an 83% expression knockdown of *THAP* (Fig. 3B) did result in a 27% and 73% decrease in *ATF-2* and *AeSCP-2* expression, respectively (Fig. 3B and Fig. 4C). The results suggest that there may be a threshold level of *THAP* transcription below which would lead to reduction in *ATF-2* and *AeSCP-2* expression in the midgut. On the other hand, a 77% reduction in *ATF-2* expression in the larval midgut (Fig. 3B) resulted in a 1.56-fold increase in *AeSCP-2* expression in the larval midgut (Fig. 5). Therefore a forward negative modulating mechanism may exist between THAP and ATF-2 in *AeSCP-2* expression regulation in the larval midgut. High levels of THAP could potentially lead to higher levels of *AeSCP-2* and *ATF-2* transcription (a positive modulating effect); the THAP-mediated up-regulation of *ATF-2* expression in turn may attenuate *AeSCP-2* transcription (Fig. 7). Expression knockdown of *THAP* led to 65% and 73% decreased in *AeSCP-2* transcript in the midgut, respectively (Fig. 5 and Fig. 4C), which is significantly less effective than the 83% reduction in *AeSCP-2* expression mediated by siAeSCP-2 (Fig. 7). It is unclear whether siTHAP-mediated 27% reduction in *ATF-2* expression (Fig. 3B) led to less efficiency in the siTHAP-mediated decreasing *AeSCP-2* transcription compare to siSCP-2 alone (Fig. 5) through the forward-negative modulation mechanism (Fig. 7). Any factor that influences the level of THAP or ATF-2 in the larval midgut would ultimately affect the overall levels of *AeSCP-2* expression (Fig. 7).

The endogenous *THAP* and *AFT-2* expression in 24 h 4^th^ instar is ubiquitous with higher levels detected in the head and carcass (Fig. 3A). It is speculated that THAP and ATF-2 have multiple target genes in different tissues at different developmental time points. Clearly, THAP and ATF-2 have the opposite effects on growth and development of *Aedes aegypti* (Fig. 3C, *F*_1,12_ = 840.5, *p*<0.0001). In this study, we focused on the THAP and ATF-2 *in vivo* function in the larval midgut regarding *AeSCP-2* promoter activity (Fig. 4), the biological effects of global expression knockdown of *THAP* and *ATF-2* on development, mortality, fertility (Fig. 3C, 3D, 6A, and 6B) may only be partially due to regulation of *AeSCP-2* in the larval midgut. A tissue-specific approach would help teasing out the specific THAP and ATF-2 function and the biological importance in the midgut tissue. Identification of the first larval midgut specific promoter (Fig. 1 and Fig. 4) should facility future studied.

In summary, results from the promoter/reporter gene assays indicate that −1.6 kb 5′ flanking sequence was sufficient for temporal/spatial regulation of *AeSCP-2* transcription in the larval midgut (Fig. 1 and 4). *AeSCP-2* is the first reported target gene of ATF-2 and THAP in mosquitoes. The results also validated the potential of using the vertical DNA delivery method for *in vivo* promoter activity studies in mosquito larvae.

## Materials and Methods

### Chemicals and Reagents

Chemicals and reagents were purchased from Sigma (St. Louis, MO), Thermo Fisher Scientific (Pittsburgh, PA) and ICN (Costa Mesa, CA) if their origins are not mentioned in the text. Enzymes for manipulating DNA during cloning processes were purchased from New England Biolabs (NEB, Ipswich, MA) or Promega (Madison, WI). Molecular biological reagents were purchased from Invitrogen (Grand Island, NY), BioRad (Hercules, CA), Qiagen (Valencia, CA).

### Mosquitoes

The yellow fever mosquito, *Aedes aegypti*, is an inbred laboratory strain (Rockefeller) that was reared at 26°C in 16 h day light/8 h night cycle in 70–80% humidity. Larvae were fed with fish food (TretraMin, Tetra Holding, Inc., Blacksburg, VA, USA). Female adults were blood fed with defibrinated rabbit blood (Hemostat Laboratories, Dixon, CA) using a glass feeder and circulation of water heated to 37°C.

### Plasmids

To construct the *hsp70* short promoter expression vector, the pBS-hsp70-SV40 poly(A) expression vector [11] was digested with *Xba*I/*Xho*I, made to be blunt-ended using Klenow^-exo^ (NEB) in the presence of 2 mM dNTPs, and then self-ligated using T4 DNA ligase (NEB), which resulted in the pBS-xhsp70-SV40 poly(A) expression vector (−194 bp 5′ flanking), the *Drosophila hsp70* short promoter. To select hairpin siRNA sequences for targeted genes, we use the online free tool from Ambion (http://www.ambion.com/techlib/misc/siRNA_finder.html). The potential target sequences was blast-searched in the EST database in VectorBase of *Aedes aegypti*, if the sequence only match perfectly to ESTs of the targeted gene without partial match to ESTs of other genes, then, we identified the siRNA as target specific sequence. DNA oligo of sense and antisense nucleotides for the small hairpin RNA targeting *THAP*, *ATF2*, *AAEL005286*, and *AAEL011794* (Table 2) was synthesized (IDT, Coralville, IA). One hundred microliters ddH_2_O was added to dissolve each primer (∼0.60 mg), 5 µl sense and antisense primer were mixed, heated at 95°C for 5 min, returned to room temperature to cool for 5 min. The annealed DNA oligo was cloned into the *Eco*RV site in MCS in pBS-xhsp70-SV40 plasmid. DNA miniprep of cloned plasmid was prepared using the Spin column kit (Qiagen). Two micrograms of plasmid DNA were digested with a restriction enzyme (recognition site inserted in the loop of the siRNA, Table 2) at 37°C for 2 h and was cleaned up with the spin column (Qiagen) before sequencing using the M13 reverse primer. DNA sequencing confirmed the hairpin insert for each gene. The plasmid of pBS-hsp70-siAeSCP2 [11] was digested with *Xba*I and self-ligated to produce the pBS-xhsp70-AeSCP2siRNA plasmid with a shortened *hsp70* promoter (−254 bp 5′ flanking *hsp70* sequence). The efficiency of heat shock induced expression knockdown was that more than 90% F0 larvae showed a significant reduction in which the target gene mRNA level was lower than the mean-S.D. in the control (Fig. S2A). Larvae were heat shocked at 37°C for 24 h on Day 1 2^nd^ and Day 4^th^ induced continuous siRNA expression through the 2^nd^ instar-pupal-adult stages (Fig. S2C).

**Table 2:** Nucleotide sequences of primers for PCR and oligos for siRNA.

Gene	Oligo*	Tm	Amplicon	Efficiency
*Rpl8*	F: 5′-TACCTGAAGGGAACCGTCAAGCAA-3′	60°C	221 bp	99.3%
	R: 5′-ACAATGGTACCTTCGGGCATCAGA-3′	60°C		
*AeAct-1*	F: 5′-CCCTGAAGTACCCCAATGAGC-3′	59°C	51 bp	99.7%
	R: 5′-CCATGTCATCCCAGTTGGTG-3′	58°C		
*AeSCP-2*	F: 5′-GCTGGTCGAGTCCGACGATGC-3′	60°C	82 bp	105.7%
	R: 5′-CAGGGCACCGGTTCCGATGG-3′	60°C		
−1.6/−1.3 kb *AeSCP-2* Probe	F: 5′-biotin-AGCTTACCTTGAATAATTAGGTACGG-3′	59°C	305 bp	
	R: 5′-biotin-AAAGGCTAAATTACCAAAAATGTAAT-3′	57°C		
*THAP*	F: 5′-ATGAGGTCAGCGAACGGGAAGAAT-3′	60°C	148 bp	95%
	R: 5′-ACGACCGGCGTTATGTTGAACTCT-3′	60°C		
*ATF-2*	F: 5′-ATGTTCTCTCGCTGCACAAGGACT-3′	60°C	191 bp	95%
	R: 5′-AGCAGCGTTAACAACAGGATTCGC-3′	60°C		
*AAEL005286*	F:5′-CAGCGATTTCCCTGCTTTCCCAAT-3′	60°C	92 bp	95%
	R:5′-AGGAGCCCTTCCTGTTCCATCAAT-3′	60°C		
*AAEL011794*	F:5′-TCGATAAGTGCTTCCGGTTCGGTT-3′	60°C	198 bp	95%
	R:5′-ACTGTTCGGTGTGGTAAGGTGGAA-3′	60°C		
−1.6 kb *AeSCP-2* promoter	F: 5′-AGCTTACCTTGAATAATTAGGTACGG-3′	55°C	1612 bp	
	R: 5′-GTCGAAACTCGAAACTGATGG-3′	54°C		
*THAP* siRNA	Sense: 5'-gatccgCGTGGAAACGAATGAGGTCtttgaattcAGACCTCATTCGTTTCCACGttttttggaaa-3'			
	Antisense: 5'-tttccaaaaaaCGTGGAAACGAATGAGGTCTgaattcaaaGACCTCATTCGTTTCCACGcggatc-3'			
*ATF-2* siRNA	Sense: 5'-gatccgCTGCCCAACAAGTCGAATCttgaattcGATTCGACTTGTTGGGCAGttttttggaaa-3			
	Antisense: 5′-tttccaaaaaaCTGCCCAACAAGTCGAATCgaattcaaGATTCGACTTGTTGGGCAGcggatc-3′			
*AAEL005286* siRNA	Sense: 5'-gatccgATTTGTCGATACAAGACCttgaattcGGTCTTGTATCGACAAATCttttttggaaa-3'			
	Antisense: 5′-tttccaaaaaaGATTTGTCGATACAAGACCgaattcaaGGTCTTGTATCGACAAATcggatc-3′			
*AAEL011794* siRNA	Sense: 5'-gatccACGCAAGCTCGGAGAATTCtttggtaccGAATTCTCCGAGCTTGCGTttttttggaaa-3'			
	Antisense: 5′-tttccaaaaaaACGCAAGCTCGGAGAATTCggtaccaaaGAATTCTCCGAGCTTGCGTggatc-3′			

* Capital letters represent sequences of perspective genes; lower case letters represent added sequence for cloning purpose or the loop in the hairpin structure.

The −1.6 kb 5′ flanking sequence of AeSCP-2 was amplified using specific PCR primers (Table 2) and the −1.6 kb/CAT construct [17] as the template in Phusion HF Master Mix reaction solution (NEB) and was cloned into the pBlunt PCR cloning vector (Invitrogene), the cloned 1.6 kb promoter was removed from pBlunt-1.6 kb via *Bam*HI/*Pst*I digestion. The *hsp70* promoter sequence was removed from pBS-hsp70-SV40 poly(A) expression vector [11] via *Bam*HI/*Pst*I digestion and the gel purified *Bam*HI/*Pst*I fragment of the 1.6 kb AeSCP-2 promoter sequence was inserted to replace the *hsp70* promoter to create the pBS-1.6 kb AeSCP-2promoter-SV40 expression vector. The *AeSCP-2* hairpin sequence was removed from the pBS-hsp70-siAeSCP-2 vector [11] using *Pst*I/*Hin*dIII and inserted into *Pst*I/*Hin*dIII sites in pBS-1.6 kbAeSCP-2promoter-SV40 vector to construct the pBS-1.6 kb-siAeSCP-2 plasmid. DNA sequencing confirmed the siAeSCP-2 hairpin insert.

*AeSCP-2* promoter/CAT constructs have been previously described [17] and were used for *in vivo* transfections. The pIE over-expression vector [17] were used to construct constitutive over-expression constructs of ATF-2 and THAP (PCR cloning primers were listed in Table 2). The over-expression vectors were sequenced to confirm the correct orientation of the inserted coding regions of ATF-2 and THAP. All plasmids used in microinjection were purified using the EndoFree plasmid Maxi Kit (Qiagen), and then filtered through 0.22 µm MCE Syringe Filter (Fisher brand, cat #09-719A). The quantity of the DNA was determined using UV OD_260_ absorption on NanoDrop (NonoDropTM1000, NanoDrop Products, Wilmington, DE, USA).

### Microinjection

The *In vivo* jetPEI (Polyplus transfection, Illkirch, France)/plasmid DNA mixture (N/P = 10) was prepared and microinjected as described previously by Peng et al. [11]. Ten female adults were microinjected with jetPEI/plasmid DNA mixture at 16–18 h post the blood meal (PBM). The amount of 0.5 µl jetPEI/plasmid DNA mixture (0.5 µg DNA plasmid/female) was injected into the hemolymph through the anterior thorax using a micromanipulator as described [11]. To obtain the consistent *in vivo* transfection results, technically 90% of the injected females should show uniformly high levels of ovarian uptake of injected DNA/PEI material from the hemolymph [11] and the mortality of microinjected adults must be <80% for each performance.

### CAT Reporter Gene Assays

*In vivo* transfected larvae were synchronized at molting to 4^th^ instar. Larval midgut and carcass was dissected in ice-cold phosphate saline buffer (PBS, pH 7.4) from 24 h 4^th^ instar larvae. Tissues (30 larvae/sample) were put separately into 500 µl 1× lysis buffer (CAT ELISA Kit, Roche Applied Science, Mannheim, Germany) containing the cocktail of protease inhibitors (Sigma). Samples were homogenized briefly using a micropestle and centrifuged at 9300×*g* for 5 min at 4°C. The amount of 200 µl supernatant was used for CAT quantity assay using the CAT ELISA kit according to the manufacturer’s instructions. The supernatant was diluted 10 times and the protein concentration for the same sample was determined using the BCA protein assay kit (Thermo Scientific Pierce, IL). A concentration standard curve (0.015–1.00 ng/ml) of pure CAT (provided in the assay kit) was constructed for each batch of assay and the amount of CAT in each sample was calculated using the CAT standard curve. CAT quantity in each sample was defined as CAT unit/min of the reaction/µg protein.

### Nuclear Protein Extraction and Boiotin-Streptavidin Pull-down Assay

Twenty four hour- and seventy two hour-old 4^th^ instar larvae were dissected in ice-cold PBS containing protease inhibitors (Protease inhibitor cocktail, Sigma) to obtain midguts (500 larvae/sample). Midguts were washed once in 500 µl ice-cold PBS/protease inhibitors and homogenized in 1 ml of hypotonic lysis buffer provided in the CelLytic NuCLEAR Extraction Kit (Sigma). The 150 µl nuclear extracts from 24 h and 72 h 4^th^ instar larval midguts were prepared according to the protocol of CelLytic NuCLEAR Extraction Kit (Sigma) and were snap-frozen using liquid nitrogen and were stored in −80°C. The protein concentration was determined using the BCA Protein Assay Kit (Thermo Scientific Pierce, IL). DNA fragment between −1.6 kb to −1.3 kb 5′ flanking sequence of the *AeSCP2* promoter was used to make the biotinylated probe for pull-down assay. Biotinylated DNA fragments were amplified by PCR. The 5′ biotin-labeled primer pairs were listed in Table 2. The amplified biotin-labeled DNA probes were gel purified and the concentration was measured using NanoDrop Spectrophotometer. Prior to use, 100 ng Dynabeads were prewashed twice with 300 µl binding buffer using magnetic separation according to the protocol (Dynabeads kilobaseBINDER™ Kit, Invitorgen). Eight hundred nanograms of biotin-labeled probes were incubated with 100 ng Dynabeads in 300 µl binding buffer (12% glycerol, 20 mM Hepes pH 7.9, 60 mM KCl, 1 mM EDTA, 1 mM dithiothreitol) at 4°C for 3 h on a roller to keep the beads in suspension. The probe-captured beads were washed 3 times in 100 µl wash buffer (15% glycerol, 20 mM Tris-HCl, pH 8.0, 1 mM EDTA). Eight hundred microgram proteins of each nuclear extract was incubated with probe-captured beads for 30 min at 4°C in 300 µl binding buffer containing 12% glycerol, 20 mM Hepes pH 7.9, 60 mM KCl, 1 mM EDTA, 1 mM dithiothreitol, and 5 µg of poly (dI-dC) non-specific competitor. The tubes were set on a roller to keep the beads in suspension. The protein-DNA-Dynabeads complex was washed 3 times with 50 µl buffer A (15% glycerol, 20 mM Tris-HCl, pH 8.0, 1 mM EDTA) containing 75 mM KCl. Bound proteins were eluted with 200 µl of buffer A (15% glycerol, 20 mM Tris-HCl pH 8.0, 1 mM EDTA) containing 1 M KCl. The eluted protein mixtures were then dialyzed in 250 ml dialysis buffer (15% glycerol, 20 mM Tris-HCl pH 8.0, 1 mM EDTA) overnight at 4°C to remove the salt.

“In Liquid” digestion and mass spectrometric analysis was conducted at the Mass Spectrometry Facility (Biotechnology Center, University of Wisconsin-Madison). In short, proteins have been extracted by precipitation with 5 times excess 10% (w/v) Trichloroacetic acid (TCA)/Acetone, incubated on ice for 30 min, centrifuged for 10 min at 16,000×*g* and pellets washed twice with ice-cold acetone, followed by once with ice-cold methanol. Pelleted proteins were re-solubilized and denatured in 20 µl of 8 M Urea/100 mM NH_4_HCO_3_ for 10 minutes then diluted to 90 µl for tryptic digestion with: 5 µl of 25 mM Dithiothreitol (DTT), 5 µl Acetonitrile (ACN), 50 µl of 50 mM NH_4_HCO_3_ and 10 µl trypsin solution (100 ng/µl Trypsin Gold from Promega Corp. in 25 mM NH_4_HCO_3_). Digestion was conducted for 2 hours at 42°C then another 10 µl of trypsin solution was added and the reaction proceeded overnight at 37°C. The digestion was terminated by acidification with 2.5% Trifluoroacetic Acid (TFA) to 0.3% final and 8 µl loaded for nanoLC-MS/MS analysis.

Peptides were analyzed by nanoLC-MS/MS using the Agilent 1100 nanoflow system (Agilent Technologies, Palo Alto, CA) connected to a hybrid linear ion trap-orbitrap mass spectrometer (LTQ-Orbitrap XL, Thermo Fisher Scientific, San Jose, CA) equipped with a nanoelectrospray ion source. Capillary HPLC was performed using an in-house fabricated column with integrated electrospray emitter essentially as described [49] but using 360 µm × 75 µm fused silica tubing. The column was packed with Jupiter 4 µm C12 particles (Phenomenex Inc., Torrance, CA) to approximately 12 cm. Sample loading (8 µl) and desalting were achieved using a trapping column in line with the autosampler (Zorbax 300SB-C18, 5 µM, 5×0.3 mm, Agilent Technologies). HPLC solvents were as follows: Isocratic loading: 1% (v/v) ACN, 0.1% Formic acid; Gradient elution: Buffer A: 0.1% formic acid in water, and Buffer B: 95% (v/v) ACN, 0.1% formic acid in water. Sample loading and desalting were done at 10 µl/min whereas gradient elution was performed at 200 nl/min and increasing %B from A of 1 to 40% in 195 minutes, 40 to 60% in 20 minutes, and 60 to 100% in 5 minutes. The LTQ-Orbitrap was set to acquire MS/MS spectra in data-dependent mode as follows: MS survey scans from m/z 300 to 2000 were collected in centroid mode at a resolving power of 100,000. MS/MS spectra were collected on the 5 most-abundant signals in each survey scan. Dynamic exclusion was employed to increase dynamic range and maximize peptide identifications. This feature excluded precursors up to 0.55 m/z below and 1.05 m/z above previously selected precursors. Precursors remained on the exclusion list for 40 sec. Singly-charged ions and ions for which the charge state could not be assigned were rejected from consideration for MS/MS. Raw MS/MS data was searched against user-defined *Aedes aegypti* amino acid sequence database (17,402 protein entries, VectorBase) using in-house Mascot search engine (Matrix Science Inc., Boston, MA, USA) with Methionine oxidation as variable modification, peptide mass tolerance was set at 20 ppm and fragment mass at 0.6 Da. Criteria to assign protein identification (annotation) was that at least two peptides with ion score greater then ∼25 or one with ion score greater than 40. Database Blast search of the predicted sequence against NCBI was performed to remove mismatches against common contaminant.

### Reverse Transcription Polymerase Chain Reaction (RT-PCR)

Quantitative analysis of AeSCP2 expression was performed by real-time quantitative RT-PCR (RT-qPCR). Staged animals were washed with ddH_2_O, rinsed once with diethylpyrocarbonate (DEPC)-H_2_O and excess water was blotted off using clean Kimwipes. For the whole body samples total RNA was extracted from individual larva or pooled 30 2^nd^ instar larvae or 10 3^rd^ or 4^th^ instar larvae or 10 pupae or adults/sample using Trizol reagent (Invitrogen). The animals from each experiment were randomly collected from each batch of transfection. Five micrograms of total RNA were treated twice at 37°C for 30 minutes with DNaseI using the Turbo DNA-free Kit (Applied Biosystems/Ambion, Austin, TX) to remove DNA contamination. RNA concentration was then measured using NanoDrop Spectrophotometer, and 0.5 µg of DNA-free total RNA was used for single-stranded cDNA synthesis using High-Capacity cDNA Archive Kit (Applied Biosystems, Austin, TX). Quantitative PCR (qPCR) was performed using the iQ™ SYBR Green Supermix (Bio-Rad Laboratories, Hercules, CA). The PCR reaction solution contained 1 µl of cDNA from the RT reaction (equivalent to 25 ng RNA). PCR was performed under the following conditions: 94°C for 3 min, 40 cycles at 94°C for 10 s, 54°C for 30 s, and a final extension at 72°C for 2 min. Primers for PCR reactions were listed in Table 2. Critical qPCR parameters of the internal control were described (Table 2). The relative mRNA levels of AeSCP-2 in RT-qPCR were compared to *Actin-2* or *rpL8* mRNA levels.

Semi-quantitative RT-PCR was performed to determine the temporal/spatial expression profiles of THAP, ATF-2, AAEL005286, and AAEL011794 in 4^th^ instar larvae. Total RNA samples were prepared from stages larvae and were made DNA-free as described above for RT-qPCR. The PCR reaction solution contained 1 µl of cDNA from the RT reaction (equivalent to 25 ng RNA) using the 2× Phusion Matster Mix (NEB). PCR was performed under the following conditions: 98°C for 3 min, 30 cycles at 98°C for 15 s, 58°C for 15 s, 72°C for 1 min, and a final extension at 72°C for 2 min. Primers for the RT-PCR were listed in Table 2. The PCR product was resolved in a 1% or 2% agarose gel in TAE buffer (40 mM Tris-acetate and 1 mM EDTA, pH 8.3) which was stained using 5 µg/ml ethidium bromide (EtBr). The gel electrophoresis image was captured using the Eagle Eye™ II Imaging System (Agilent Technologies, Santa Clara, CA).

### Western Blotting Analysis

Intact pupae selected from transfected groups (siTHAP/SCP-2EGFP and SCP-2EGFP) were washed with ddH_2_O and the excess water was blotted off with a clean Kimwipe (KIMTECH, Dallas, TX). Western blotting analysis was performed as described [29] using SDS 4–20% gradient PAGE gel (ISC BioExpress, Kaysville, UT).

### Statistical Analysis

Data were analyzed with two-way ANOVA (GLM procedure) to determine if several components of the biological parameter in the control groups and treated groups differed significantly using the GraphPad PRISM software version 4.0 (GraphPad). Unpaired t-test with Welch's correction was performed for two groups with unequal numbers of samples to determine whether the differences were significant using the GraphPad PRISM software version 4.0 (GraphPad). Student’s t-test was used in cases where a pair of treatments with equal numbers of samples was compared to determine the significance of the differences [50].

## Supporting Information

Figure S1 **Western Blotting analysis of AeSCP-2 expression in transfected pupae.** Lane 1: Protein molecular weight markers; lane 2: siTHAP/SCP-2EGFP co-transfected male pupae; lane 3: siTHAP/SCP-2EGFP co-transfected female pupae; lane 4: siRNA vector/SCP-2EGFP transfected male pupae; lane 5: siRNA vector/SCP-2EGFP transfected female pupae. Total soluble proteins (20 µg/lane) were resolved on 4–20% gradient SDS PAGE gel. Affinity purified rabbit anti-AeSCP-2 antibody (1∶1000 dilution) and horse reddish peroxidase (HRP) conjugated goat anti-rabbit antibody (1∶2000 dilution) were used.
(DOC)

Figure S2 **Effects of duration of heat shock on** ***AeSCP-2*** **siRNA** ***in vivo*** **expression driven by the short** ***Drosophila*** **hsp70 promoter (M&M). Relative** ***AeSCP-2*** **mRNA levels (vs.** ***rpL8*** **) were determined via RT-qPCR.** (**A**) Day 1 F0 4^th^ instar larvae were heat shocked for indicated durations and the total RNA from each randomly selected 15 individuals was extracted as described (M&M). Bars = mean and standard deviation (N = 15). (**B**) Synchronized Day 1 2^nd^ instar larvae were heat shocked at 37°C for 24 hours and returned to 26°C for the rest of the growth till samples (10 per sample) were taken. (**C**) Larvae were synchronized on Day 1 2^nd^ instar, heat shocked for 24 hours on Day 1 of 2^nd^ returned to 26°C until Day 1 4^th^ instar. A 2^nd^ heat shock-treatment at 37°C for 24 hours was applied to Day 1 4^th^ instar, and then returned to 26°C for the rest of the growth. Day 1 4^th^ samples (10 larvae/sample) were taken after the second heat shock treatment. Pupae and adult samples were mixed sexes. Mean and standard deviation are shown (N = 3). Heat-shock for 24 hours on Day 1 of 2^nd^ and 4^th^ instar, respectively, led to significant expression knockdown of the target gene throughout the entire 2^nd^ instar to adult development.
(DOC)
