# Supplementary material for: THAP and ATF-2 Regulated Sterol Carrier Protein-2 Promoter Activities in the Larval Midgut of the Yellow Fever Mosquito, Aedes aegypti
Source: PLoS One. 2012 Oct 4;7(10):e46948. doi: 10.1371/journal.pone.0046948 (PMC3464256; doi:10.1371/journal.pone.0046948)
Supplement: Figure S1 — Western Blotting analysis of AeSCP-2 expression in transfected pupae. Lane 1: Protein molecular weight markers; lane 2: siTHAP/SCP-2EGFP co-transfected male pupae; lane 3: siTHAP/SCP-2EGFP co-transfected female pupae; lane 4: siRNA vector/SCP-2EGFP transfected male pupae; lane 5: siRNA vector/SCP-2EGFP transfected female pupae. Total soluble proteins (20 µg/lane) were resolved on 4–20% gradient SDS PAGE gel. Affinity purified rabbit anti-AeSCP-2 antibody (1∶1000 dilution) and horse reddish peroxidase (HRP) conjugated goat anti-rabbit antibody (1∶2000 dilution) were used. (DOC) [file pone.0046948.s001.doc]

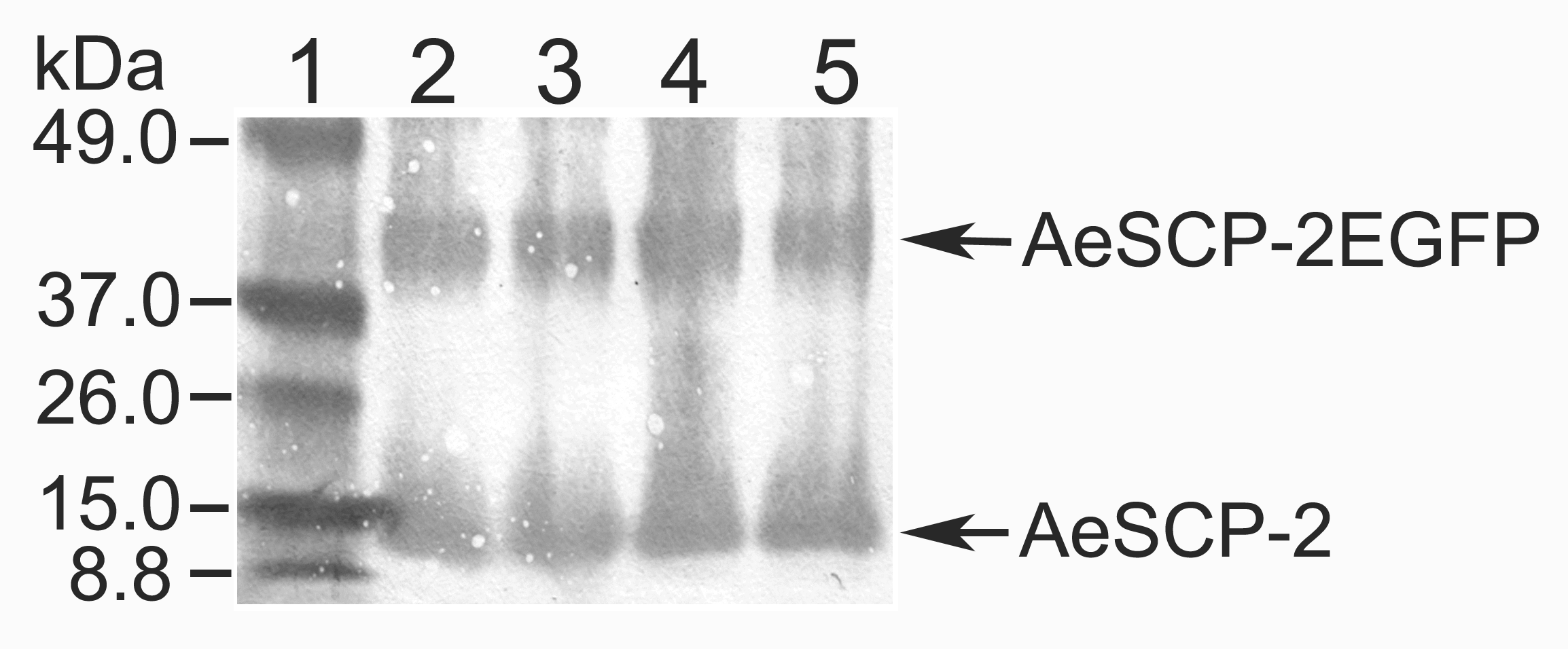


**Supplementary Figure 1**. Western Blotting analysis of AeSCP-2 expression in transfected pupae. Lane 1: Protein molecular weight markers; lanes 2 and 4: siTHAP/SCP-2EGFP co-transfected male pupae; lanes 3 and 5: siTHAP/SCP-2EGFP co-transfected female pupae. Total soluble proteins (20 μg/lane) were resolved on 4-20% gradient SDS PAGE gel. Affinity purified rabbit anti-AeSCP-2 antibody (1:1000 dilution) and horse reddish peroxidase (HRP) conjugated goat anti-rabbit antibody (1:2000 dilution) were used.
