# Supplementary figures and images for: THAP and ATF-2 Regulated Sterol Carrier Protein-2 Promoter Activities in the Larval Midgut of the Yellow Fever Mosquito, Aedes aegypti
Source: PLoS One. 2012 Oct 4;7(10):e46948. doi: 10.1371/journal.pone.0046948 (PMC3464256; doi:10.1371/journal.pone.0046948)

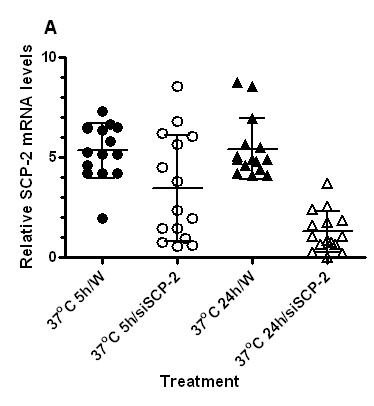

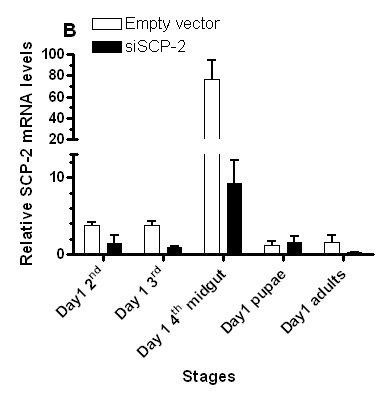

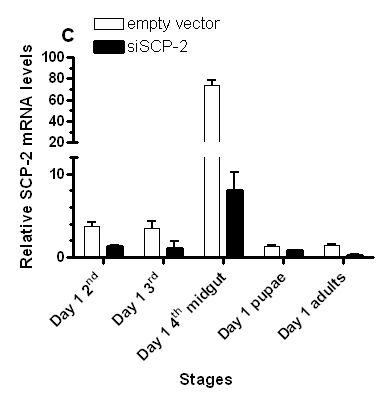


**Supplementary Figure 2**.

Supplement: Figure S2 — Effects of duration of heat shock on AeSCP-2 siRNA in vivo expression driven by the short Drosophila hsp70 promoter (M&M). Relative AeSCP-2 mRNA levels (vs. rpL8 ) were determined via RT-qPCR. (A) Day 1 F0 4th instar larvae were heat shocked for indicated durations and the total RNA from each randomly selected 15 individuals was extracted as described (M&M). Bars = mean and standard deviation (N = 15). (B) Synchronized Day 1 2nd instar larvae were heat shocked at 37°C for 24 hours and returned to 26°C for the rest of the growth till samples (10 per sample) were taken. (C) Larvae were synchronized on Day 1 2nd instar, heat shocked for 24 hours on Day 1 of 2nd returned to 26°C until Day 1 4th instar. A 2nd heat shock-treatment at 37°C for 24 hours was applied to Day 1 4th instar, and then returned to 26°C for the rest of the growth. Day 1 4th samples (10 larvae/sample) were taken after the second heat shock treatment. Pupae and adult samples were mixed sexes. Mean and standard deviation are shown (N = 3). Heat-shock for 24 hours on Day 1 of 2nd and 4th instar, respectively, led to significant expression knockdown of the target gene throughout the entire 2nd instar to adult development. (DOC) [file pone.0046948.s002.doc]
